# Epigenome-wide epidemiologic studies of human immunodeficiency virus infection, treatment, and disease progression

**DOI:** 10.1186/s13148-022-01230-w

**Published:** 2022-01-11

**Authors:** Boghuma K. Titanji, Marta Gwinn, Vincent C. Marconi, Yan V. Sun

**Affiliations:** 1 Division of Infectious Diseases, Emory University School of Medicine, Atlanta, GA USA; 2 Department of Epidemiology, Rollins School of Public Health, Emory University, 1518 Clifton Road NE #3049, Atlanta, GA 30322 USA; 3 Atlanta Veterans Affairs Health Care System, Decatur, GA USA; 4 Hubert Department of Global Health, Rollins School of Public Health, Atlanta, GA USA; 5 Emory Vaccine Center, Yerkes National Primate Research Center, Emory University, Atlanta, GA USA

**Keywords:** EWAS, HIV, Aging, Chronic diseases

## Abstract

Despite significant advances in the treatment and care of people with HIV (PWH), several challenges remain in our understanding of disease pathogenesis to improve patient care. HIV infection can modify the host epigenome and as such can impact disease progression, as well as the molecular processes driving non-AIDS comorbidities in PWH. Epigenetic epidemiologic studies including epigenome-wide association studies (EWAS) offer a unique set of tools to expand our understanding of HIV disease and to identify novel strategies applicable to treatment and diagnosis in this patient population. In this review, we summarize the current state of knowledge from epigenetic epidemiologic studies of PWH, identify the main challenges of this approach, and highlight future directions for the field. Emerging epigenetic epidemiologic studies of PWH can expand our understanding of HIV infection and health outcomes, improve scientific validity through collaboration and replication, and increase the coverage of diverse populations affected by the global HIV pandemic. Through this review, we hope to highlight the potential of EWAS as a tool for HIV research and to engage more investigators to explore its application to important research questions.

## Introduction

Last year marked the 40th anniversary of the first reported cases of acquired immunodeficiency syndrome (AIDS). In the last four decades, considerable progress has been made in our understanding of the biology of human immunodeficiency virus (HIV), the causal agent of AIDS. Antiretroviral therapy (ART) has transformed what was once a deadly infection into a chronic and manageable disease. The availability of effective preventative measures has led to a steady decline of new HIV cases globally, and there is renewed hope for vaccine development and HIV cure research.

Despite these significant advances, several challenges remain for people with HIV (PWH). The burden of non-AIDS related disease conditions (NADC) associated with chronic inflammation attributed to HIV, accelerated aging, and immune non-response (INR) while virologically suppressed on ART, continue to have a significant negative impact on quality of life and longevity for PWH. There is a need for innovative approaches to address these new challenges and identify effective interventions. This review summarizes the state of knowledge derived from epigenome-wide association studies (EWAS) of HIV infection, treatment, and progression while exploring the role of EWAS as a key approach to addressing emerging challenges in HIV research.

## A foundational primer on epigenetic modifications

Epigenetic information consists of modifications of nuclear DNA or histones that are maintained during cell division but are also influenced by environmental factors [1]. There are three major forms of epigenetic information: DNA methylation, histone modification, and chromatin conformation, all of which can regulate gene expression [2].

*DNA methylation (DNAm*) is the addition of methyl (CH_3_)-groups to DNA which can modify the function of genes and their expression. One common form is the methylation of the fifth carbon of the pyrimidine ring of a cytosine in the cytosine-guanine dinucleotide (known as CpG sites). In mammalian cells up to 70% of CpG sites are methylated [3], a process which can be influenced by intrinsic and extrinsic host factors, leading to alterations in how genes are expressed and regulated.

*Histone modification—*Histones are proteins associated with DNA in the nucleus and a key component of the chromatin. They have a role in condensing DNA into chromatin and can undergo post-translational modifications via multiple mechanisms: acetylation, methylation, phosphorylation, ubiquitylation, and sumoylation. These modifications can alter three-dimensional chromatin structure and impact gene expression.

*Chromatin conformation—*Chromatin is a complex of protein and nucleic acids (RNA and DNA) that makes up genes in eukaryotic cells. Chromatin can undergo modification leading to conformational changes that impact gene expression. Changes in chromatin remodeling proteins and binding RNAs have been associated with human malignancies and other diseases.

**Fig. 1:**
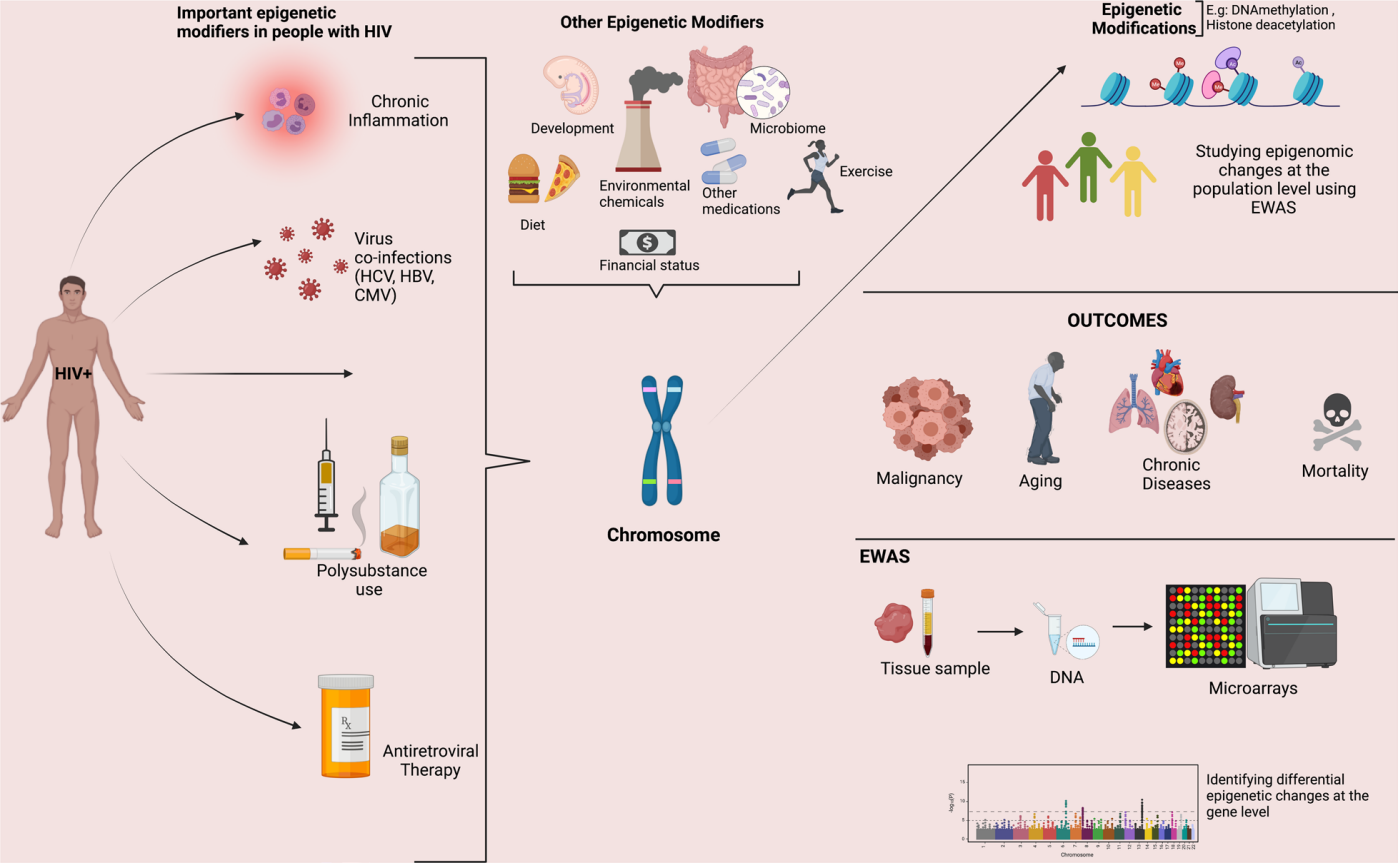
Factors leading to epigenetic modifications in people with HIV and the downstream consequences of these changes. Abbreviations: *DNA* deoxyribonucleic acid, *EWAS* epigenome-wide association study, *HCV* hepatitis C virus, *HBV* hepatitis B virus, *CMV* cytomegalovirus, *HIV* human immunodeficiency virus

In the setting of HIV infection, epigenetic modifications can be mediated by the virus itself or by the inflammatory processes triggered by chronic viral infection. Subsequently, epigenetic modification can increase the expression of pro-inflammatory cytokine genes resulting from infection, further exacerbating the inflammatory cascade. Details of the complex pathways implicated in these processes are beyond the scope of the current review but have been summarized by others [4, 5]. Figure 1 illustrates the factors contributing to epigenetic modifications in PWH.

## Key challenges in HIV research

### Chronic inflammation and biomarkers

Chronic inflammation and immune activation are well recognized as major drivers of pathogenesis and disease in PWH [6]. They persist even with effective suppression of viral replication and have been implicated in NADCs and frailty in this population. Several markers of inflammation such as interleukin-6 (Il-6), soluble cluster determinant-14 (sCD14) and tumor necrosis factor-alpha (TNF-α) are persistently elevated in PWH and are significantly associated with disease progression and mortality [6]. At present, few pharmacological interventions are effective in reducing inflammation in PWH. Although EWAS may offer insights into the mechanisms of inflammation and immune activation in HIV, these insights might not lead directly to new therapeutic strategies because epigenetic changes could be a secondary effect or byproduct of chronic inflammation. Potential causal roles for epigenetic markers require further investigation (e.g., by Mendelian randomization [7]).

### Non-AIDS related disease conditions (NADCs)

Compared with the general population, PWH have a disproportionately higher burden of chronic diseases, termed NADCs. HIV infection is an independent risk factor for cardiovascular disease (CVD) and PWH experience higher incidence of stroke, myocardial infarction, and coronary artery disease much earlier in life than their aged-matched HIV-negative counterparts [8]. Chronic kidney disease (CKD) is also a frequent complication of HIV infection, occurring in 3.5% to 48.5% of PWH depending on the cohorts studied [9]. CKD is mediated by factors related to HIV infection, genetic predisposition, ART, environmental factors and other chronic medical conditions like diabetes mellitus and hypertension. Understanding how these factors interact to promote CKD in PWH is crucial for identifying ways to mitigate this co-morbidity.

ART does not fully restore immune function in PWH and, despite the declining incidence of AIDS-defining malignancies the incidence of many other cancers, remains high [10, 11]. This risk is further enhanced by the higher prevalence of concomitant oncogenic virus infections (Epstein Barr virus (EBV), human herpes virus-8 (HHV8), human papilloma virus (HPV), hepatitis B virus (HBV) and hepatitis C virus (HCV)) [12] and a higher prevalence of tobacco and other substance use in this patient population [12]. Chronic antigen stimulation from longstanding HIV infection results in T and B cell exhaustion, loss of antiviral effector function, and impaired antibody production; all of these factors contribute to reduced viral clearance, impaired immune tumor surveillance, and increased risk for malignancy [12]. Increased longevity extends the cumulative exposure to chronic inflammation, oncogenic viral co-infections, and carcinogens, and the accumulation of somatic mutations and epigenetic changes related to carcinogenesis [10, 11].

Impaired cognitive function is the main manifestation of HIV neurologic involvement and remains a challenge for PWH [13]. When compared to healthy HIV-negative controls, around half of PWH continue to have lower levels of performance on neuro-psychometric testing than would be predicted [13]. There is a paucity of reliable biomarkers for diagnosis of HIV-associated neurocognitive disorders (HAND) or prediction of neurologic decline. Although non-specific, neopterin can be a useful biomarker for HIV-associated dementia. Neopterin is a bioproduct of the guanosine triphosphate pathway, produced primarily in monocyte/macrophage-related cells [14]. No therapeutic interventions have been found to improve the symptoms of HAND.

Chronic pulmonary disease [15] and liver disease [16] are also more prevalent in PWH as are contributing factors such as illicit drug use, tobacco abuse, obesity, and co-infection with other viral pathogens. Epidemiologic studies that incorporate epigenetic markers can help elucidate the interactions between these contributing factors and HIV infection, as well as the independent and joint contributions of individual contributing factors to NADC in PWH.

## HIV and epigenetic changes

DNAm of host genes in response to HIV-1 infection has been implicated in mechanisms of viral latency, HIV-1 transcription, and viral replication [17–19]. As an example, HIV-1 can trigger methylation of host genes directly by inducing the DNA methyl transferase-1 enzyme in vitro [20]. Host epigenetic regulatory machinery can control proviral DNA by cellular epigenetic regulators which may modify the state of chromatin, thus modulating HIV latency and reactivation [20]. A detailed description of the mechanisms by which HIV modulates epigenetic changes in the host genome is beyond the scope of this review but has been summarized by others [20].

Epigenetic reprogramming is therefore able to regulate not only the life cycle of the virus at multiple stages, but also the interaction between the host and the virus genome with implications for disease pathogenesis and clinical outcomes. Epigenome-wide association studies (EWAS) draw on bioinformatic and statistical tools to identify epigenetic markers that are associated with phenotypes at the population level. In this review, we focus on DNAm because it is the only epigenetic marker that has been analyzed in population-based studies of PWH.

## Design and workflow of EWAS in PWH

EWAS in PWH most often employ retrospective case–control, cross-sectional, or longitudinal designs, as illustrated by the studies highlighted in this review. Retrospective case–control studies typically compare epigenetic markers in cases (HIV-positive) and controls (HIV-negative) who have the phenotype of interest. Cross-sectional studies may or may not include HIV-negative controls and only provide a snapshot of epigenetic modifications relative to the phenotype of interest at one point in time. Retrospective case–control and cross-sectional studies are limited by the fact that they cannot determine the direction of causation of the epigenetic changes observed. Furthermore, cross-sectional studies do not allow a complete exploration of how the relationship between HIV, ART, and DNAm may change over time. Longitudinal studies follow a cohort of HIV-positive individuals over time with repeated tissue sampling. By identifying epigenetic changes prospectively, longitudinal studies provide a stronger basis for causal inference and overcome some of the limitations of retrospective and cross-sectional study designs. This design has been applied in DNAm age-acceleration studies as well as in pre-ART and post-ART studies of PWH.

**Fig. 2:**
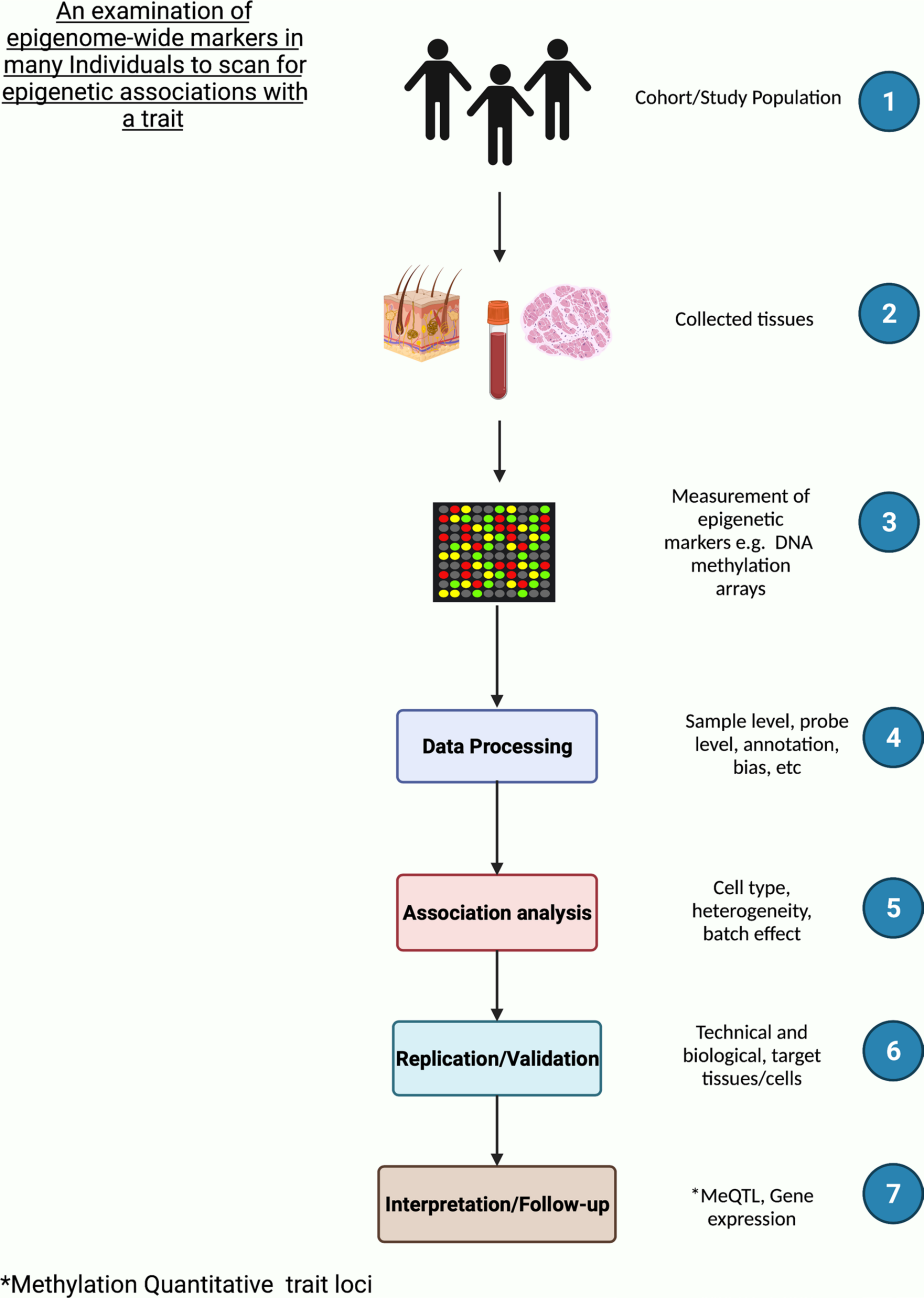
The typical workflow of EWAS

The choice of tissue for sampling is crucial for all EWAS but is generally constrained by accessibility. Most EWAS in PWH are based on DNAm in blood samples due to the challenges of obtaining relevant tissues; for example, brain tissue biopsy is rarely feasible for studies of HIV-associated neurocognitive disease. Blood samples may not always be biologically relevant to the phenotype of interest. Furthermore, using blood samples as a surrogate requires careful interpretation due to variable cell-type composition. Figure 2 summarizes the typical workflow of EWAS.

## Epigenetic epidemiologic studies of PWH

Epigenetic epidemiologic research on HIV infection, progression, and outcomes is still at an early stage. Several key published findings so far are based on the Veterans Aging Cohort Study (VACS), a study of patients with and without HIV infection who are seen in infectious disease and general medical clinics operated by the Veterans Health Administration [21]. Other studies of PWH have collected similar DNAm data and begun to explore the role of DNAm on the disease risk and health span among PWH [20, 22–26].

### HIV-infection and viremia

In a study of 186 predominantly male US veterans, Zhang and colleagues examined differentially methylated CpG sites in the host genome of PWH and HIV-negative individuals to determine whether HIV-associated DNAm sites correlated with viral load [27]. Twenty sites were found to be differentially methylated in PWH compared to the controls without HIV, three of which showed a significant association with viral load. In this study, hypomethylation of two CpG sites (cg07839457 and cg16411857) in the promoter of the *NLRC5* gene was associated with HIV infection and replicated in an independent sample. *NLRC5* plays a role in antiviral immunity and the cytokine response by inhibiting the transcription factor nuclear factor kappa beta (NF-KB). Methylation changes in *NLRC5* may be associated with uncontrolled HIV replication through upregulating NF-KB and interferon-I (IFN-I) signaling pathways. The top three CpG sites significantly associated with HIV infection were located near or within genes related to immune activation, which is a central feature of chronic HIV infection and disease pathogenesis.

### Antiretroviral therapy

Although ART reduces HIV replication, it does not fully eliminate the virus from the host due to the persistence of latently infected immune cells. In a small cohort of 11 PWH on ART with fully suppressed viremia (< 50 copies/mL), extremely low levels of methylated CpG dinucleotides were observed within the HIV 5′ LTR of resting CD4^+^ T cells [28]. In contrast, another study found that in six PWH on ART without detectable viremia, CpG methylation of the HIV 5′ LTR was 20%, 30%, 48%, 71%, 96%, and 100%—compared with < 0.1% in a control group of viremic patients [29]. These discordant findings suggest that factors such as subtypes of CD4^+^ T cells [30], history of infection, duration and type of ART, degrees of turnover of latently infected CD4^+^ T cells, and inclusion of unintegrated HIV DNA may play a significant role in the variation of DNAm, viral persistence, and latency.

A pediatric cohort study of South African children compared DNAm profiles of 120 children with HIV on ART with suppressed viremia to those of 60 age-matched children without HIV. This study identified 1309 differentially methylated CpG sites in children with HIV compared to their age-matched controls [26]. Strong differential methylation in the same region of the *NLRC5* gene identified in adult cohorts of PWH was also found in this pediatric cohort. In contrast to a study based on the adult male VACS cohort that identified hypomethylated sites in individuals with HIV compared to individuals without HIV, 97% of DNAm CpG sites identified in children with HIV were hypermethylated with respect to age-matched controls [26]. This suggests that exposure to HIV infection and ART in early life may impact the epigenome differently from exposure later in life, which could have implications for differential disease manifestations in children compared to adults.

In EWAS of PWH on ART with fully suppressed viremia, it is challenging to separate the effects of ART exposure and HIV infection on the epigenome. In addition, concurrent factors such as chronological aging, environmental factors, and other infectious exposures may also leave marks on the epigenome. Furthermore, cross-sectional studies do not allow a complete exploration of how the relationship between HIV, ART, and DNAm may change over time; thus, the directionality of the relationship cannot be inferred (i.e., inability to separate cause from effect). Longitudinal studies may be more informative in both pediatric and adult populations. Most studies to date have used the Illumina Human Methylation 450 K BeadChip Array (Illumina Inc, San Diego, CA), which only covers 1.5% of genomic CpG sites and is biased towards promoter and protein coding regions. Additional studies using the more recent EPIC 850 K array (Illumina Inc. San Diego, CA) and DNA methylation sequencing, which offers broader coverage in other genomic regions, are needed to provide a more comprehensive view.

### Chronic diseases and HIV

PWH carry a significant burden of chronic diseases impacting multiple end organs, including the kidneys, lungs, brain, heart, and liver. The underlying molecular processes that explain this increased disease burden are incompletely understood. EWAS has the potential to unlock clues into disease pathogenesis and identify new therapeutic targets. In recent years this powerful tool has been exploited to examine the association between DNAm and several chronic conditions in PWH. Table 1 provides a summary of EWAS of chronic diseases, co-morbid factors and HIV.

**Table 1:** Summary of DNAm EWAS in cohorts of PWH

Outcomes	Study design and characteristics of the study population	Platform	Key findings
DNAm in HIV+ individuals with viremia [27]	Case–control study viremic HIV+ individuals compared to HIV-negative controls US veterans, predominantly male *N* = 186	450 K	20 differentially methylated sites identified in the HIV+ cohort compared to the HIV^−^ controls Hypomethylation of CpGs in promoter of NLRC5 gene associated with HIV infection
DNAm of HIV 5’ LTR in HIV+ individuals on suppressive ART [28]	Cross-sectional study aviremic HIV+ individuals on suppressive ART *N* = 11	Targeted	Low levels of methylated CpGs within the HIV 5’ LTR
DNAm of HIV 5’ LTR of HIV+ individuals on suppressive ART [29]	Case control study aviremic individuals on ART compared to control group of viremic patients *N* = 6	Targeted	High levels of DNAm in aviremic patients compared to < 0.1% of CpGs pf HIV-1 promoters in viremic patients
DNAm profile HIV+ children with suppressed viral load [26]	Case control study HIV+ children on ART with undetectable VL and matched HIV- controls *N* = 180	450 K	1309 differentially methylates CpGs in HIV+ children compared to age-matched HIV- controls Strong differential methylation in the region of NLRC5 gene as seen in adult cohorts
Association between blood DNAm and lung function in HIV+ [22]	Cohort study HIV+ adults, detectable VL, not on ART and CD4 counts > 500, > 40 years old *N* = 161	850 K	Airflow obstruction defined by FEV1/FVC < 0.7 associated with differential DNAm
Association between DNAm changes and eGFR in HIV+ individuals [31]	Case control study All male participants HIV + individuals and HIV- controls	450 K	15 differentially methylated CpG sites associated with eGFR in HIV + individuals
DNAm of PBMCs in HIV+ individuals with cognitive impairment [33]	Case control study HIV+ individuals and HIV^−^ controls *N* = 31	450 K	1032 differentially methylated CpG sites associated with CI in HIV+ individuals compared to HIV^−^ controls
Epigenetic association with T2DM by HIV status and interaction effects [36]	Case control study HIV+ individuals and HIV^−^ controls *N* = 681	450 K	Identification of several CpG sites in HIV+ individuals associated with T2DM
Association between EWAS of smoking and HIV prognosis and mortality [45]	Retrospective cohort 1137 HIV+ individuals	450 K and 850 K	Identification of 137 CpGs associated with smoking in PWH. DNAm CpGs also shown to be predictive of prognosis and mortality in PWH
Mediation role of DNAm sites on the effect of cocaine use on HIV severity [48]	Longitudinal cohort study HIV+ veterans *N* = 1081	450 K	Identification of 12 differentially methylated CpG sites with significant mediation effects
Interacting epigenetic factors in HIV+ individuals with HCV co-infection and IVDU [53]	Case control study HIV+ individuals with HCV and IVDU and HCV^−^/IVDU^−^ controls *N* = 798	450 K	Significant methylation differences in genomes of HIV+/IVDU+/HCV+ individuals compared to controls
Patterns of DNAm associated with progression of HIV infection [55]	Case control study HIV+ elite controllers, HIV+ individuals on ART with detectable VL, aviremic HIV+ individuals on ART, HIV- controls *N* = 85 samples from 64 individuals	850 K	Differential methylation in CpG sites in viremic HIV+ individuals compared to HIV- controls, between viremic and aviremic individuals on ART and viremic individuals and elite controllers. No differences in DNAm in elite controllers compared to HIV^−^ individuals
Association between DNAm and mortality in HIV+ individuals [56]	Cohort study HIV+ veterans form the VACS cohort *N* = 1081	450 K	393 CpG sites identified with excellent performance for predicting mortality risk in HIV+ individuals with high VACS score

*LTR* long terminal repeat, *HIV* human immunodeficiency virus, *VACS* Veterans Aging Cohort Study, *DNA* deoxyribonucleic acid, *m-DNAm* DNA methylation, *T2DM* type 2 diabetes mellitus, *VL* viral load, *HCV* hepatitis C virus, *CI* cognitive impairment, *IVDU* intravenous drug use, *PWH* people with HIV, *FEV* forced expiratory volume, *FVC* forced vital capacity, *ART* antiretroviral therapy, *EWAS* epigenome-wide association study

#### Chronic pulmonary disease

Significantly higher rates of chronic obstructive pulmonary disease (COPD) and overall pulmonary function decline have been observed in PWH [15]. It remains unclear whether individual factors such as smoking, illicit drug use, recurrent infections, chronic inflammation, or a combination of these factors drive the increased risk for COPD in PWH. Cordero et al. tested the association between blood DNAm and lung function in a cohort of 161 PWH [22]. They assessed pulmonary function parameters including forced expiratory volume in one second (FEV_1_), forced vital capacity (FVC), the FEV_1_/FVC ratio, and the decline in FEV_1_ over a period of 5 years. They found that 1393 differentially DNAm sites were associated with airflow obstruction defined as FEV_1_/FVC < 0.70 while 4676 DNAm sites were associated with airflow obstruction defined as FEV1/FVC < lower limit of normal. Overall, airflow obstruction was associated with global hypomethylation. DNAm associations were not found with FEV_1_ decline.

Interestingly, the Cordero et al. study found that the DNAm sites associated with airflow obstruction were enriched for biological pathways associated with chronic viral infections, such as hepatitis B, Epstein Barr virus, and human papilloma virus [22]. This observation suggests a potential role for viral co-infections such as chronic herpes viruses and hepatitis viruses, which are more prevalent in cohorts of PWH, as contributors to differential DNAm associations with pulmonary function parameters. This study cohort was restricted to individuals > 40 years with detectable viral load and CD4^+^ T cell counts > 500 cells/mL, who were not on ART at the time of entry. It is therefore difficult to say whether these findings would apply to PWH on ART for many years, who have achieved virologic suppression. Furthermore, the proportion of the cohort meeting criteria for airway obstruction was small (*n* = 20), and larger cohorts will be needed to confirm these findings.

#### Kidney disease

HIV infection is associated with an increased risk for chronic kidney disease (CKD), defined as a reduced estimated glomerular filtration rate (eGFR) < 60 mL/min/1.73 m^2^. A study of 567 men with HIV and 117 men without HIV in the Veterans Aging Cohort Study (VACS) explored epigenetic changes related to eGFR in PWH [31]. Using the Illumina 450 K array to survey peripheral blood mononuclear cells, 15 CpG sites were significantly associated with eGFR among the HIV-positive participants. The three most significant CpG sites (located at *MAD1L1*, *TSNARE1/BAI1*, and *LTV1*) were all negatively associated with eGFR. This study attempted to replicate CpG associations with eGFR reported in previous non-HIV EWAS with limited success, although directionality of the associations was consistent. This could reflect limited statistical power and potential differences between people with and without HIV infection. They also profiled DNAm in peripheral blood cells rather than in kidney tissue, which is more relevant for CKD. This is a limitation of many such studies in understanding functional mechanisms of target organs.

#### HIV-associated neurocognitive disorders (HAND)

HIV-associated cognitive impairment (CI) is characterized by a wide range of clinical manifestations ranging from mild cognitive decline to debilitating behavioral changes, motor function decline, and dementia. The use of ART has dramatically reduced the prevalence of HAND, but less severe forms continue to occur in 40% of PWH on treatment [23]. Monocytes/macrophages contribute to the pathogenesis of HIV-associated CI [32], but considerable gaps remain in our understanding of the underlying mechanisms.

Corley et al. examined DNAm of peripheral blood mononuclear cells (PBMCs) from 21 PWH who met clinical criteria for CI in the Hawaii HIV Aging Cohort Study for comparison with 10 individuals with HIV and normal cognitive function [33]. They identified 1032 CI-associated, differentially methylated loci in monocytes and observed CI-associated methylation differences linked to gene expression (assessed by targeted human transcriptome profiling) and neuropsychological test scores. They also noted a 10% difference in methylation at specific loci (occurring preferentially at regulatory regions of the genome, including CpG island shores, gene bodies, intergenic, and enhancer regions) related to genes involved in the CNS and interacting with HIV. These findings support the role of epigenetic perturbations of monocytes in HIV-associated CI and the potential to identify immuno-epigenetic signatures that could be used to research novel therapeutic approaches. The results of this small study still need to be validated in larger cohorts, preferably with pure subsets of monocytes such as microglia, which play a vital role in brain dysfunction [34].

#### Diabetes mellitus

Factors that increase the risk of developing type 2 diabetes mellitus (T2DM) in PWH include long-term survival from HIV infection and use of ART, hepatitis C virus (HCV) co-infection, and elevated BMI [35]. Epigenetic modifications of DNA are independently associated with HIV infection and with T2DM; the co-existence of both create the possibility of interaction effects in studies of DNAm and disease outcomes. Mathur et al. investigated epigenetic associations with T2DM according to HIV infection status and assessed interaction effects among 681 male participants from the Veterans Aging Cohort Study [36]. They replicated the previously reported association of cg19693031 (*TXNIP*) with T2DM, demonstrating a stronger association in the HIV^−^positive than in the HIV-negative group. *TXNIP* has also been shown to be associated with several inflammatory markers in a cohort of HIV-negative individuals [37]. They also identified several T2DM-associated CpG sites (cg1231141 (*ADAMTS2*), cg19534769 (*HGFAC*), and cg13163919 (*TLE3*) implicated in inflammation, pancreatic β-cell function, and T2DM pathogenesis [36] in PWH for further investigation.

### Co-morbid factors and HIV

#### Smoking and HIV infection

Several studies indicate that the prevalence of smoking in PWH is 2–3 times higher than in the general population [38–43]. Smoking in PWH is associated with increased mortality and amplifies the risk for other chronic, co-morbid conditions in this population [44]. Zhang et al. applied ensemble machine learning to identify smoking-related DNA methylation signatures that were predictive of prognosis and mortality of HIV infection [45]. The study population included 1137 PWH in VACS, divided into a discovery sample (361 smokers and 247 non-smokers) and a validation sample (309 smokers and 220 non-smokers). They performed a meta-EWAS of the discovery and validation samples and identified 137 CpGs associated with smoking [45]. To examine whether smoking-associated CpGs were predictive of HIV frailty and mortality, machine learning was utilized to build a model employing 408,583 CpGs. 698 CpGs were predictive of high HIV frailty and a DNAm index constructed from these CpGs was associated with a hazard for mortality [45]. In this retrospective study, smoking status was defined by self-report, which could introduce bias. These findings will need to be confirmed in prospective cohorts.

#### Cocaine use and HIV infection

Cocaine use is more common in PWH than in the general population and has been associated with HIV disease progression [46] by mechanisms that are not fully understood. Some studies suggest that substance abuse disorders may be associated with inadequate adherence to ART [47]; however, cocaine use also causes epigenetic changes that could mediate the impact of cocaine use on HIV progression and disease severity. A study of 1435 veterans with HIV in VACS examined DNAm as a potential mediator of the association of cocaine use with HIV severity [48]. In this study, cocaine use was associated with a higher VACS index (a validated measure of frailty/mortality) and with increased mortality, findings consistent with previously studied cohorts [21, 49]. They identified 12 differentially methylated CpG sites in genes involved in the antiviral response (*IFIT3, IFITM1, NLRC5, PLSCR1, PARP9*) and in HIV progression (*CX3CR1*, *MX1*) [48]. These CpG sites also showed significant mediation effects, individually explaining between 11.3 and 29.5% of the effect of persistent cocaine use on HIV severity. Strengths of this study included the availability of longitudinal data, clearly defined measures of cocaine use, and an unbiased EWAS approach; however, the study was limited by the predominantly male cohort and a small sample size used for mediation analyses.

#### Intravenous drug use (IVDU) and HCV co-infection

Both IVDU and HCV infection may worsen HIV outcomes by amplifying chronic inflammation and immune activation [50–52]. A study of PWH in VACS found that PBMC-based DNAm significantly different between IVDU^+^/HCV^+^ and IVDU^−^/HCV^−^ persons, which could be linked with HIV outcomes measured by the VACS index [53]. Methylated regions associated with IVDU^+^/HCV^+^ status were located on genes important for mediating antiviral immunity and inflammation [53]. These findings suggest that the link between methylation markers and HIV outcomes in IVDU^+^ /HCV^+^ individuals may be related to immune activation, which is strongly associated with HIV progression, comorbidity, and frailty. Differential DNA methylation associated with IVDU and HCV may worsen HIV outcomes by regulating gene expression of immune and inflammatory proteins. The main limitation of this study was the inability of the investigators to separate IVDU from HCV infection because these exposures were tightly linked.

### HIV disease progression and mortality

DNA methylation profiles predictive of mortality have been identified in general population cohorts [54]. DNAm changes mediated by HIV infection have the potential to modulate both disease progression and mortality in PWH. An EWAS conducted by Moron-Lopez and colleagues identified distinct patterns of CD4^+^ T-cell DNAm associated with HIV disease progression [55]. They analyzed 85 samples from 64 participants, including 21 elite-controllers, 21 individuals with viremia before ART and under suppressive ART (sampled before ART initiation and viremic suppression and after ART initiation with undetectable viral load), and 22 HIV-negative donors. They found that viremic individuals had 129 methylated CpG sites that were significantly different compared with HIV-negative individuals, 162 CpG sites compared with treated individuals, and 441 CpG sites (163 gene promoters) compared with elite controllers. No differences were found between elite controllers and HIV-negative individuals. The tumor necrosis factor (TNF) promoter region was hypermethylated and correlated with elevated plasma levels of TNF in viremic persons compared with the ART-receiving, elite controller and HIV-negative groups [55]. Several studies have demonstrated the role of TNF in the pathogenesis of HIV with effects on various stages of viral replication, including the ability of TNF to increase HIV transcription through activation of the NF-κB signaling pathway. The small number of participants in this study limited its ability evaluate potential differential effects of specific antiviral agents on DNAm.

Another group applied machine learning to EWAS data from the VACS cohort, using the ensemble prediction model to identify a panel of DNAm features associated with mortality in PWH. They included 1081 HIV-positive individuals from VACS who were divided into subsets for training (*n* = 460), validating (*n* = 114) and testing (*n* = 507) the model [56]. The VACS index [49] was applied as measure of mortality risk among PWH. A prediction model using 393 CpG sites showed excellent performance in predicting 10-year mortality. The biological relevance of these 393 CpG sites was supported by gene ontology enrichment analyses [56]. Top enriched pathways were associated with viral response, immune activation, and cytokine receptor binding, which could contribute to the increased risk of mortality among PWH. This study highlights the applicability of machine learning to EWAS data as a way of identifying new biological markers of disease and predictors of disease outcome. The specific prediction model, however, has limited generalizability because all data were from middle-aged men with HIV.

## DNAm as a marker of aging for PWH

**Fig. 3:**
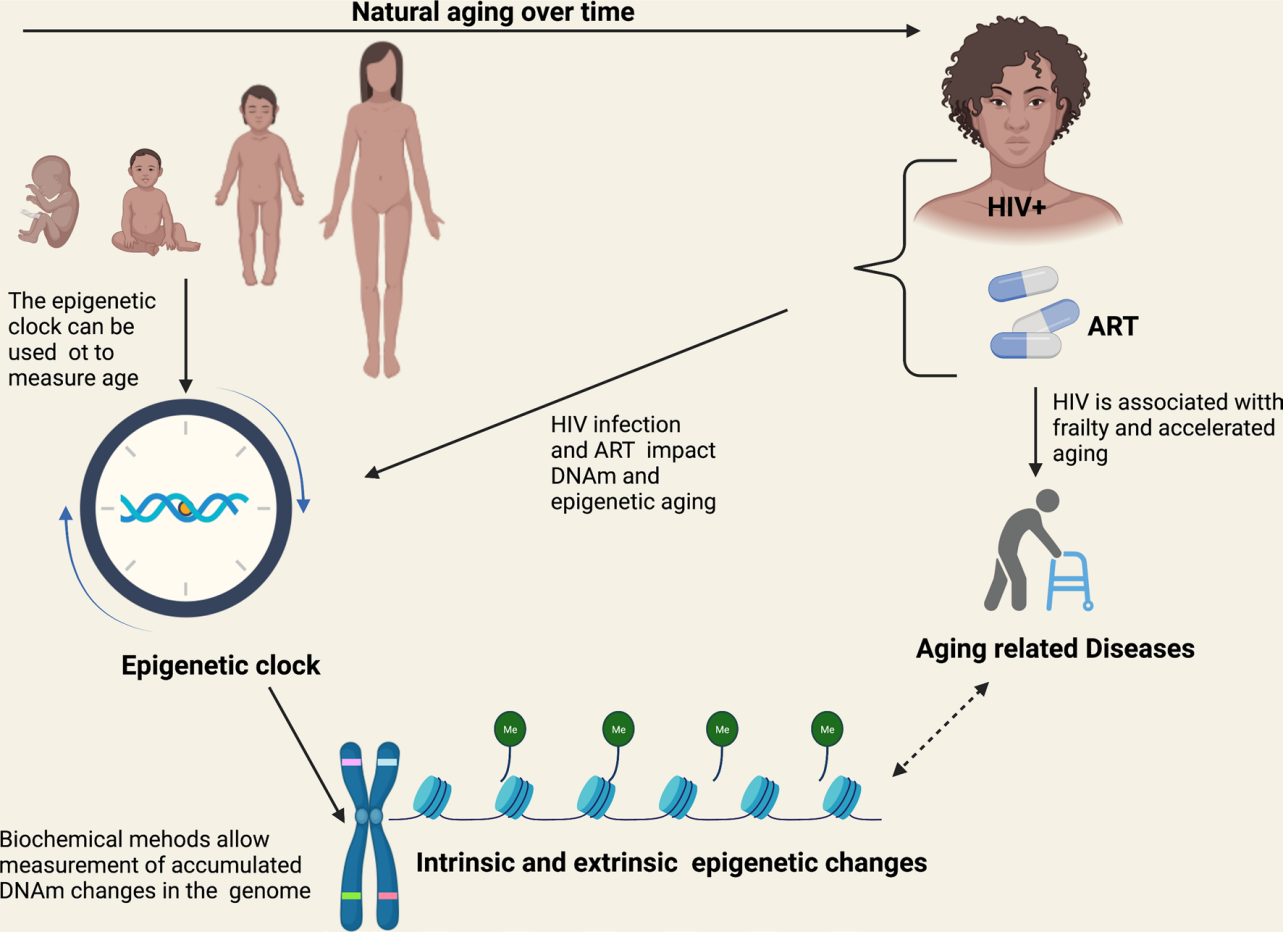
DNA methylation and aging in People with HIV. Abbreviations: *ART* antiretroviral therapy, *DNA* deoxyribonucleic acid, *DNAm* DNA methylation, *HIV* human immunodeficiency virus

In 2018, more than half (51%) of PWH were aged 50 and older and this proportion was expected to rise [57]. HIV infection may contribute significantly to early onset aging through multiple mechanisms. HIV can lead to DNA damage through direct and indirect mechanisms [58]. Telomere shortening has been well described in cohorts of PWH and has been associated with aging though causation has not been firmly established [59, 60]. Disruption of homeostasis between protein synthesis and degradation increases with aging and is associated with chronic diseases which tend to be overrepresented in PWH [61, 62]. Mitochondrial dysfunction, which could be associated with HIV infection itself or with certain antiretrovirals used for treatment, may contribute to accumulation of mutations in the mitochondrial DNA and promote premature aging [63]. Chronic, low grade HIV replication may also contribute to cellular senescence (reviewed in [64]). Figure 3 illustrates important interactions and the factors contributing to DNA methylation and aging in PWH.

At the molecular level, aging has been associated with epigenetic modifications including DNA methylation, histone modification and chromatin remodeling [65]. Epigenetic tools have been developed to predict epigenetic age based on DNAm changes (reviewed in [65]). Epigenetic age is also known as DNAm age and is calculated by applying mathematical algorithms to a selection of aging-related DNAm sites [65]. Several studies have shown associations between aging and DNAm in the genome [66–68].The most frequently used approach, the Horvath clock, estimates age, based on 353 DNAm epigenetic markers in the genome. The basic approach is to form a weighted average of the 353 clock CpGs, which is then transformed to DNAm age using a calibration function [65]. Table 2 provides a summary of EWAS and aging in PWH.

**Table 2:** Summary of DNA methylation aging studies in PWH

Outcomes	Study design and characteristics of the study population	Platform	Key findings
Epigenetic aging based on DNAm markers of aging [69]	Case control study 8 data sets, brain specimens and blood specimens of PWH and matched HIV negative controls	450 K and 27 K	Increased epigenetic aging in brain tissue (7.4 years) and in blood (5.2 years) of PWH compared to HIV negative controls
Epigenetic aging based on DNAm markers of aging [70]	Case control study 137 HIV+ individuals compared to 44 HIV negative controls	450 K	4.9-year aging advancement in HIV+ individuals compared to HIV-negative controls. Increased expected mortality of 19% in PWH
Epigenetic aging based on DNAm and other markers of epigenetic aging [71]	Case control study 12 HIV+ individuals and 12 HIV- controls	450 K	Age advancement of 14 years in in HIV+ individuals compared to HIV- controls
Epigenetic aging based on DNAm and epigenetic clock [72]	Case control study 378 ART naïve HIV+ individuals with CD4 counts > 500 compared to 34 HIV- controls	850 K	Accelerated aging advancement occurs in PWH with preserved immune function compared to HIV- controls
Epigenetic aging based on DNAm and other epigenetic markers of age acceleration [26]	Case control study Pediatric cohort HIV+, HIV-exposed uninfected (HEU) and HIV unexposed, uninfected (HUU) children *N* = 178	450 K	No differences in age acceleration between the three groups. However, significantly shorter telomere length in HIV^+^ children compared to HEU and HUU groups
Epigenetic aging based on DNAm markers of aging [25]	Case control study 31 perinatally infected HIV+ individuals compared to 30 HIV-negative controls	850 K	Differences in epigenetic age acceleration and extrinsic epigenetic age acceleration between HIV^+^ and HIV-negative groups. No difference in intrinsic epigenetic aging between groups
Epigenetic aging based on DNAm markers of aging [74]	Longitudinal cohort study 19 HIV+ individuals compared to 19 HIV-negative controls	450 K	Baseline DNAm age of PWH prior to ART initiation 11.2 years greater than HIV-negative controls
Epigenetic aging based on DNAm based markers of aging and epigenetic clock [75]	Prospective cohort study 4-year follow up period 63 aviremic HIV^+^ individuals on ART	850 K	No acceleration of epigenetic aging in the cohort over the follow-up period
Epigenetic aging based on DNAm markers of aging and epigenetic clocks [78]	Longitudinal cohort study 168 HIV+ individuals before ART initiation and 2 years after ART initiation compared to 44 HIV-negative controls with similar age and sex distribution	850 K	Epigenetic age acceleration in HIV+ individuals compared to HIV-negative controls. Reversal in epigenetic aging after 2 years of ART
Inflammation related single nucleotide polymorphisms as risk factors for age [80] acceleration and NADCs	Post-mortem cohort HIV+ individuals *N* = 155	450 K	Epigenetic aging significantly greater in IL-6 CC carriers and IL-10 CC homozygotes compared to other genotype groups
Association between DNAm markers of aging with measures of cognitive function in PWH [24]	Case control study 69 HIV+ individuals and 38 HIV-negative controls. Cohort > 60 years	850 K	Significant negative correlations between intrinsic epigenetic aging and executive function, attention and working memory and PhenoAge and attention

*IL* interleukin, *HIV* human immunodeficiency virus, *ART* antiretroviral therapy, *EWAS* epigenome-wide association study, *NADC* non-AIDS related disease conditions

Using DNAm as an epigenetic marker for aging, Horvath and Levine reported that HIV infection led to accelerated epigenetic aging in both brain tissue (7.4 years) and blood (5.2 years), demonstrating the potential of the epigenetic clock as a tool for studying aging related NADCs in PWH [69]. Another study compared 137 people with HIV on sustained ART with 44 controls without HIV and found that both chronic and recent HIV infection led, on average, to a 4.9-year increase in DNAm age and increased expected mortality by 19% [70]. Rickabaugh, in a smaller study, compared 12 individuals with HIV not on ART with 12 controls without HIV and reported an epigenetic age increase of 14 years [71]. This discrepancy with the other two studies could reflect either the beneficial effects of ART or the different statistical approaches applied. A more recent study compared 378 ART-naive persons with HIV having CD4^+^ T-cell counts > 500/µL with 34 controls without HIV, calculating DNAm age using the epigenetic clock [72]. They found that in PWH not on ART, even with preserved immune function, there is evidence of advanced DNAm aging. These results suggest that the aging process potentially starts early during HIV infection and substantial methylation changes can occur even in the absence of advanced disease and AIDS defining conditions. There is some indication that ART can help mitigate these changes and temper age acceleration but this would need to be confirmed in studies of larger cohorts, as should the utility of DNAm age as a biomarker in the care of PWH.

Interestingly these findings were not confirmed in a pediatric cohort of children with HIV in South Africa. Shiau et al*.* conducted a cross-sectional study of 120 children including HIV-positive, HIV-exposed uninfected (HEU) and HIV-negative unexposed uninfected (HUU). After adjusting for differences in cell-type proportions, they found no differences in DNAm associated with age acceleration between children with HIV and the other groups [26]. Telomere length was however noted to be significantly shorter in children with HIV compared to children in the HEU and HUU groups. Several factors may explain the difference in findings between this pediatric cohort and studies in adults. It is possible that the negative impact of HIV on DNAm age is mitigated by initiation of ART soon after birth in children who are perinatally infected, or that it occurs only after a longer duration of infection (i.e., greater than the mean of 6.4 years observed in this study) or later in life. Prospective cohorts with follow-up from perinatal age through adolescence and adulthood are needed to clarify the relationship of HIV infection with DNAm age in children.

A cross-sectional study that compared 31 perinatally infected young African American adults aged 20–35 years with 30 HIV-negative individuals found significant differences in two markers of epigenetic age acceleration (EAA)—intrinsic (IEAA) and extrinsic (EEAA)—between HIV-positive and HIV-negative groups [25]. EEAA is a measure of DNAm age that directly incorporates age-related changes in blood cell composition [73]. There was no difference in IEAA*—*a measure of DNAm independent of blood cell composition between the groups. In the HIV-positive individuals, EAA and EEAA were higher in those with HIV viral load ≥ 50 copies/mL compared to individuals with undetectable viral load (< 50 copies/mL). Negative correlations were also observed between EEAA and executive function, attention, and language scores [25]. Ninety percent of participants with HIV had initiated ART perinatally and had controlled viremia [25]. Early ART treatment and virologic suppression may explain the absence of a difference in IEAA between HIV-positive and the HIV-negative controls but a definitive conclusion is limited by the small size of the study as well as the absence of longitudinal data.

Using the VACS cohort, Nelson et al. studied DNAm age in 19 ART-naïve men with HIV compared to 19 HIV-negative controls at baseline before ART initiation and 7–11 years later [74]. They found that DNAm age in men with HIV was on average 11.2 years higher than HIV negative control participants at baseline; two HIV-positive men in the cohort showed a recovery in accelerated DNAm age after ART initiation [74]. The main limitation of the study was its small sample size and the lack of female participants; however, studying DNAm age in PWH prior to ART initiation allowed the authors to examine the methylation effects of HIV infection itself, rather than the combination of HIV infection and therapy.

Esteban-Cantos et al. conducted a larger longitudinal study of epigenetic aging in 63 adults with HIV who were well-controlled on ART; during a 4-year follow-up period, they found no evidence for EAA [75]. Longitudinal changes in measures of epigenetic aging were independent of the ART regimen, CD4^+^ T-cell count, and other factors related to HIV infection. The main strength of this study is that it prospectively evaluated epigenetic age acceleration in a population of long-term viremic adults with HIV using three different measures of epigenetic aging (Horvath´s clock and the estimators PhenoAge and GrimAge) [75]. PhenoAge is an epigenetic aging surrogate marker which incorporates multiple measures such as chronological age, lymphocyte percentage, albumin and glucose levels [76]. GrimAge is another multifactorial marker of epigenetic aging which incorporates chronological age, sex, smoking and several DNAm estimators of plasma proteins [77]. Both PhenoAge and GrimAge have been applied to predicting mortality and age-related changes in blood cell composition [77].This study was however limited by the lack of a control group without HIV and the lack of sufficient power to detect distinctive differences in epigenetic age acceleration with different classes of ART.

Another recently published study [78], a sub-analysis of participants from the NEAT001/ANRS143 trial [79], looked at DNAm aging in PWH before and after ART initiation compared to an HIV-negative control group with similar sex and age distribution. DNAm was measured using frozen whole blood samples from 168 HIV-positive individuals before ART and two years after ART) [78]. Epigenetic age acceleration was assessed using 4 estimators (Horvath’s clock, Hannum’s clock, GrimAge and PhenoAge). A CD4 + T-cell count < 200 cells/uL and detectable viral load > 100,000 copies/mL at baseline were associated with more pronounced epigenetic aging. Epigenetic age acceleration was reduced after two years on ART though PhenoAge and GrimAge remained higher in the HIV-positive group compared to HIV-negative controls. No significant differences in epigenetic aging were noted between the two treatment regimens [78]. These important findings support the role of ART in reversing epigenetic aging. Only two ART regimens were assessed in this study. Other ART regimens may have more profound effects on reversing epigenetic age acceleration, which can be addressed in future studies with larger and more diverse samples.

Another study of 155 post-mortem examinations of PWH investigated whether three inflammation-related single nucleotide polymorphisms (SNPs) were risk factors for accelerated aging and NADCs [80]. The authors found that epigenetic aging (higher Z-score) was significantly greater in IL-6 C-allele carriers and IL-10 CC homozygotes compared to other genotype groups. TNF genotype was not associated with epigenetic aging or NADCs. IL-6 is a pro-inflammatory cytokine associated with increased mortality in PWH [81]. The processes of accelerated aging in HIV are intimately linked to chronic inflammation and immune activation, which are hallmarks of HIV infection. Chronic inflammation promotes tissue damage and epigenetic modification. Studies such as this may provide insight into the pathophysiology of accelerated aging in PWH.

More recently, studies of epigenetic aging have been extended as a way of identifying markers of disease. A study of an African American (AA) cohort including 69 PWH and 38 HIV negative controls, all over 60 years old, estimated the association of six DNAm markers of aging with measures of cognitive function (the NIH cognition toolbox and the Montreal Cognitive assessment tool) [24]. In PWH, intrinsic epigenetic age acceleration was negatively correlated with executive function, attention, and working memory.

## Main challenges and future directions

The nascent field of EWAS applied to HIV biology and epidemiology holds potential that has yet to be harnessed. Our review discusses a growing body of studies in this field. Several challenges remain in interpreting current findings and their potential implications for improving the lives of PWH:

- The variety of array platforms used (old and new) and heterogeneous sample characteristics in studies published to date makes it challenging to compare findings between studies and limits the ability to validate interesting observations.
- The cross-sectional design of most studies, though convenient, only provides a snapshot at a point in time of epigenetic changes and may not capture the dynamic nature of this process.
- Additional limitations in many studies include small sample sizes, the lack of control groups (both individuals without HIV infection and individuals with HIV infection on ART with virologic control), and the ability to stratify by type of ART.
- For studies of epigenetic aging, the variety of measures and estimators makes it challenging to compare results between studies. There is a need for a universal standard for assessing DNAm age.
- EWAS is a useful tool for generating hypotheses linking genes and markers to disease pathogenesis and therapeutic targets; however, only a limited number of these leads have been further evaluated in biological studies.
- The associations between DNAm, chronic diseases, and aging in PWH hint at an important role of epigenetic modifications in the pathogenesis of HIV infection and warrant functional studies to fully understand the implications for disease progression and clinical outcomes.
- EWAS is limited by the tissue sampled. For studies in PWH, this has been mostly blood cell subsets, which are easy to obtain but which may be less relevant in capturing meaningful epigenetic modifications for other tissues such as the brain, kidneys, lungs liver etc. It remains unclear whether epigenetic modifications identified in blood cells are relevant surrogates for organ-specific disease processes.
- The important confounding issue of whether epigenetic modifications are a cause or a consequence of disease processes remains a challenge.

Future EWAS studies applied to larger patient cohorts with appropriate controls and longitudinal study designs will address some of the main limitations of existing studies. Using newer array platforms with more genomic coverage will allow a more comprehensive assessment of epigenetic modifications by exploring a wider range of genes relevant to HIV disease. Finally, tissue-specific sampling for EWAS either through post-mortem studies or biopsies of relevant tissue will allow for more relevant data on epigenetic modification in organ-specific disease processes.

Combining epigenetic studies with other approaches to studying gene function and expression, such as RNA sequencing and proteomics, could help expand our understanding of the role of gene expression in the development of human disease. Additional epigenetic epidemiologic studies of PWH will help inform research on HIV infection and health outcomes, improve scientific validity through collaboration and replication, and encompass more of the diverse populations affected by the global HIV pandemic. The intersection of HIV and epigenetics research remains a vast space with enormous potential for impact. We hope to engage more investigators in incorporating this approach to existing and emerging questions in the field of HIV research.

## Data Availability

Not applicable.
